# Facteurs thérapeutiques affectant la cicatrisation au cours des gangrènes du périnée

**DOI:** 10.11604/pamj.2018.29.70.14669

**Published:** 2018-01-24

**Authors:** Oussama Baraket, Wissem Triki, Karim Ayed, Sonia Ben Hmida, Mohamed Amine Lahmidi, Abdelamjid Baccar, Samy Bouchoucha

**Affiliations:** 1 Service de Chirurgie Générale, Hôpital Habib Bougatfa de Bizerte, Tunisie; 2 Faculté de Médecine de Tunis, Université Tunis El Manar, Tunis; 3 Service de Gastro Entérologie, Hôpital Habib Bougatfa de Bizerte, Tunisie

**Keywords:** Gangrène du périnée, chirurgie, cicatrisation, pronostic, Gangrene of the perineum, surgery, healing, prognosis

## Abstract

Les gangrène du Fournier est une fasciite nécrosante rare et grave. Elle est grevée d'une lourde morbi-mortalité nécessitant une prise en charge médico-chirurgicale rapide et énergique. Le traitement initial repose sur la réanimation associée au débridement chirurgical. Secondairement le problème majeur reste la cicatrisation et les séquelles que gardent les patients. Plusieurs modalités thérapeutiques sont actuellement disponibles a fin d'améliorer et accélérer la cicatrisation. Notre étude rétrospective a colligé 20 cas. L'âge médian dans notre série était d 56 ans. Il y avait 16 hommes et 7 femmes. Une comorbidité était présente chez 15 patients. L'antibiothérapie a été entreprise dans tous les cas avec une durée médiane de 15 jours Le traitement chirurgical était entrepris dans tous les cas. Des révisions itératives étaient nécessaires chez tous les patients avec une médiane de 3 changements de pansements. Une colostomie était réalisée dans 6 cas. Une oxygénothérapie hyperbare était réalisée dans 4 cas. Une vaco-thérapie dans 1 cas. Un geste de recouvrement cutané était nécessaire dans 2 cas. La durée médiane de cicatrisation était de 15 jours avec l oxygénothérapie versus 24 jours en l absence de ce traitement. La durée médiane d'hospitalisation était de 20 jours. Quatre décès ont été dénombrés dans cette série. L obtention d'une cicatrisation sans séquelles est un chalenge dans la prise en charge thérapeutique. Malgré l'adjonction de nouvelles modalités thérapeutique ses résultats restent non satisfaisants. Cependant une approche multidisciplinaire associée à une oxygénothérapie et une vac-thérapie pourrait améliorer les résultats chez ces patients.

## Abstract (en)

Fournier gangrene is a rare and severe necrotizing fasciitis. It is burdened with high morbi-mortality, requiring early and massive medical and surgical management. Initial treatment is based on patient's resuscitation associated with surgical debridement. Subsequently, the main challenge is the healing process and its possible sequelae. Several therapeutic approaches are currently available to improve and accelerate the healing process. We conducted a retrospective study of 20 cases. The median age of our patients was 56 years. The study included 16 men and 7 women. Comorbidity was present in 15 patients. Antibiotic therapy was administered in all cases, with a median duration of 15 days. All patients underwent surgery. Iterative reviews were necessary in all patients, who needed, on average, 3 dressing changes. Colostomy was performed in 6 cases. Hyperbaric oxygen therapy was performed in 4 cases. Vacuum assisted closure was performed in 1 case. Soft tissue coverage was necessary in 2 cases. The mean duration of healing was 15 days with oxygen therapy versus 24 days in the absence of this treatment. The mean duration of hospitalization was 20 days. Four patients died. Healing process without sequelae is a therapeutic challenge. Despite the addition of new therapeutic approaches, outcomes are not satisfactory. However, multidisciplinary approach associated with oxygen therapy and vacuum assisted closure might improve patients outcomes.

## Introduction

La gangrène de fournier est une dermo-hypodermite bactérienne nécrosante et grave des régions périnéales décrite pour la première fois par Baurienne en 1764, mais c'est Fournier qui donna son nom à la maladie en décrivant, en 1883, cinq cas survenant chez des hommes jeunes, de gangrène du scrotum [1]. Il s'agit d'une fasciite nécrosante péri anale, périnéale et génitale qui résulte d'une infection poly microbienne dont la source peut être génito-urinaire, colorectale, cutanée ou idiopathique et qui est potentiellement létale. Par ailleurs, elle est la cause la plus fréquente de perte de substance de peau génitale. Le diagnostic est clinique, les investigations paracliniques permettent un diagnostic précoce et une évaluation des retentissements anatomiques et biologiques. Malgré les progrès des techniques et des modalités de l'anesthésie réanimation la mortalité reste élevée avoisinant les 20à 30% [2, 3]. La prise en charge thérapeutique comporte 3 volets: un volet médical basée sur la réanimation et l'antibiothérapie; un premier volet chirurgical reposant sur le débridement chirurgical, la mise à plat des collections et des fusées purulents ainsi que l'excision de tous les tissus nécrosés et dévitalisées. Cette excision doit être la plus large possible. Ainsi le 3^ème^ volet et le volet de la cicatrisation et le recouvrement cutané. Cette cicatrisation repose sur plusieurs modalités thérapeutique: cicatrisation dirigée, oxygénothérapie hyperbare, vaco thérapie lambeaux et gestes de recouvrement [4, 5]. Le but de notre travail est de rapporter notre expérience en matière de gangrène périnéale en insistant sur les facteurs affectant la rapidité et la qualité de la cicatrisation.

## Méthodes

Etude rétrospective descriptive colligeant 22 patients pris en charge pour gangrène de fournier au service de chirurgie générale de l'hôpital Habib Bougatfa de Bizerte Sur une période de 8 ans entre 2008 et 2016. Nous avons inclus tous les patients qui ont présenté une gangrène du périnée quelque soit la cause initiale périnéale de l'abcès de la marge anale) urologique ou gynécologique. Nous n'avons pas inclus les patients qui présentaient des suppurations périnéales sans véritable gangrène. Les patients qui ont été rapidement transférés vers d'autres services. Le recueil de données a été effectué par un canevas comportant 45 variables concernant les données épidémiologiques des patients, les données cliniques, les modalités thérapeutiques et l'évolution.

## Résultats

L âge médian dans notre série était de 56 ans avec des extrêmes entre 28 et 90 ans. Il y avait 16 hommes et 7 femmes. Une comorbidité était présente chez 15 patients. Il s'agissait principalement de diabète dans 14 cas d'une HTA dans 5 cas et d'une comorbidité cardiaque dans 3 cas. Un patient était suivi d'une recto colite hémorragique et une patiente présentait un cancer des poumons avec des métastases osseuses. Cinq patients avaient plusieurs comorbidités. Le délai moyen entre le début de symptômes et l'admission était de 12 jours (entre 8 et 25 jours). 12 de nos patients avaient été mis sous antibiotiques avant la 1ère consultation en chirurgie. La douleur et la fièvre ont été les symptômes généraux les plus retrouvés (36 patients soit 80% des cas). Une hyperleucocytose était retrouvée dans 21 cas; un diabète déséquilibré chez 14 patients. L'origine de la gangrène était périnéale (abcès de la marge annale) dans 13 cas; urologique dans 6 cas; gynécologique dans 2 cas et compliquant une brulure du périnée dans 1 cas. Un scanner abdominal était pratiqué dans 2 cas. L'antibiothérapie a été entreprise dans tous les cas. La durée moyenne de l'antibiothérapie était de 15 jours. Le traitement chirurgical était entrepris dans tous les cas. Il s agissait d'une nécrosectomie avec débridement chirurgical dans tous les cas. Des révisions itératives sous anesthésie générale étaient nécessaires dans tous les cas avec une médiane de 4 avec des extrêmes de 2 à 8. Ailleurs des changements de pansements quotidiens étaient effectués. Une colostomie latérale sur baguette était réalisée dans 6 cas. Elle était terminalisée dans 5 cas. La colostomie était réalisée lors de la première intervention dans 4 cas et secondairement dans 2 cas. Le drainage urinaire était effectué chez tous nos patients. Il était effectué par sondage trans urétral dans 21 cas et par un cathéter sus pubien dans un cas. Quatre décès ont été observés dans notre série (18%). Les causes de décès étaient un choc septique réfractaire dans 3 cas et une embolie pulmonaire massive dans 1 cas. Une oxygénothérapie hyperbare était effectuée dans 5 cas. A enlever la répétition: le délai de cicatrisation était de 12 jours en cas d'oxygénothérapie versus 24 jours en l'absence de ce traitement. La durée médiane d'hospitalisation était de 20 jours avec des extrêmes de 15 à 42 jours. Un geste de recouvrement ultérieur par un lambeau était effectué dans 2 cas. Il s'agit d'une greffe de peau totale dans 1 cas et un lambeau musculo-cutané dans 1 cas.

## Discussion

La gangrène de Fouiner est une fasciite nécrosante rapidement progressive du périnée et des organes génitaux externes qui entraîne une nécrose étendue des tissus mous [2]. Il s agit de la complication la plus grave des suppurations de la région péri anale quel qu'on soit l'infection primitive. La prise en charge thérapeutique de la gangrène périnéale comporte deux volets essentiels: en urgence il faut juguler le sepsis et enlever la nécrose; à distance assurer une cicatrisation la plus complète possible avec les moindres séquelles. Le pronostic et le délai de cicatrisation dépend essentiellement de la qualité de la prise en charge mais aussi de l'étendue de la nécrose. Les autres éléments qui conditionnent le pronostic étant le terrain du patient et l'origine de la gangrène [3]. En effet dans notre série 15 patient étaient classés ASA2 ou plus. Il faut réaliser de larges excisions afin de faire l'exérèse de la totalité des tissus infectés et dévitalisés. Le décollement tissulaire induit par les germes anaérobies aide souvent à trouver le plan d´exérèse. Il faut effondrer toutes les logettes au doigt, exciser l'ensemble des tissus aux ciseaux ou au bistouri jusqu'à retrouver un fascia d'aspect macroscopiquement sain qui l'objectif principal du premier temps chirurgical. Les excisions itératives sont souvent nécessaires. Elle était de 5 en moyenne dans la série d'Ettalbi [6]. La place de la colostomie reste à déterminer. Cependant celle-ci reste largement utilisée dans la littérature (60-100% des cas). Son principal objectif est de dériver les matières fécales permettant d'éviter la souillure des pertes de substances [4, 7, 8]. Ceci permet d'accélérer le temps de cicatrisation. Dans la série de Cymzec [7] la mortalité était plus élevée dans le groupe de patients ayant une colostomie. La colostomie est pratiquée systématiquement chez tous les malades par certains auteurs [9]. D autres la préconisent chez des patients sélectionnés notamment les gangrènes à point de départ ano-rectal et en cas d'atteinte sphinctérienne [6, 8]. L'oxygénothérapie hyperbare est utilisée de plus en plus dans la prise en charge secondaire des gangrènes périnéales [3, 4, 10]. Des l'obtention d'un état local satisfaisant plusieurs auteurs la recommandent. Ses effets bénéfiques passent par une augmentation de la concentration locale en oxygène, elle améliore ainsi la fonction leucocytaire, facilitant la cicatrisation et empêchant la multiplication des bactéries anaérobies [11, 12].

Cependant son utilisation n'est pas de pratique courante notamment dans les pays en voie de développement telle que la Tunisie en raison de sa non disponibilité. Le deuxième problème se heurte l'oxygénothérapie hyperbare est les contre indications d'ordre cardiaque et respiratoire. en effet dans l'expérience de boyer [11], ce traitement était réalisée chez les 3/4 des patients (81 patients), contre indiquée chez 15 patients, et interrompue chez 11 patients en raison d'effets secondaires (essentiellement instabilité hémodynamique et épilepsie). Dans notre expérience elle a été utilisée dans 4 cas. L'évolution était favorable chez tous ces patients avec un délai de cicatrisation plus rapide qu'en l'absence de son utilisation. Dans une étude, récente portant sur 34 malades la mortalité était de 12,5% en utilisant l'oxygénothérapie hyperbare versus 21% en L'absence de ce procédé [10]. Dans une large série de 344 patients [13] portants sur les fascéites nécrosantes tout venant avec 33% de gangrène périnéale; L'oxygénothérapie diminuait de façon statistiquement significative la mortalité (12 versus 34%). En analyse multivariée elle est retrouvée avec l'âge et les comorbidités comme un facteur prédictif indépendant de réduction de la mortalité avec un odds ratio de 0.42 IC (0.22-0.83), et un p à 0.01. Des résultats similaires étaient retrouvés dans d'autres séries [14, 15]. Malgré ses résultats encourageants sa place exacte dans la littérature reste à déterminer. En effet Il n'existe pas d'étude randomisée montrant l'efficacité de l'oxy-génothérapie hyperbare dans la gangrène de Fournier après la chirurgie associée à l'antibiothérapie. Le VAC^®^ (KCI, San Antonio, Texas), système aspiratif à pression négative, est une alternative aux pansements classiques permet par son système aspiratif d'éliminer les éléments infectieux de la plaie et favorise la cicatrisation en permettant la formation rapide de tissu de granulation [16-18]. Le Vác thérapie semble donner de bons résultats. Ces limites principales sont ces contre indications notamment plaies tumorales, d'exposition des vaisseaux et/ou nerfs, ou d'important lymphoedème. Le deuxième inconvénient est le cout élevé de cette technique. A fin du palier au problème du cout l'utilisation du modèle Vacuum Pack modifié de Parker peut etre une alternative interssante. En plus le faible effectif des séries rapportant l'expérience avec la vac thérapie rend difficile l'interprétation des résultats et ne permet pas de définir sa place exacte dans la prise en charge thérapeutique. L'association OHB et Vac thérapie a été tenté par certains auteurs et semble donner de bons résultats notament dans l'étude de Janane qui rapporte une faible mortalité de 11% [17]. Les gestes de recouvrement cutané peuvent s'avérer nécessaire en cas de perte de substance cutané étendue. Pour les pertes de substances moyennement étendue on a souvent recours à des greffes de peau [19, 20]. La greffe de peau mince est préconisée pour le pertes de substance modérée mais elle est pourvoyeuses de rétraction cutanés et de sequelles douloureuses. Des lambeau musculo-cutané, le plus souvent d´un lambeau utilisant le muscle droit interne, plus rarement le muscle gracile sont nécessaires en cas de perte de substance cutané importante [4].

## Conclusion

La gangrène de Fournier est une urgence médico-chirurgicale majeure. La prise en charge doit être pluridisciplinaire car elle associe un traitement chirurgical agressif, une antibiothérapie adaptée au spectre du type d'infection en cause qu'il faut bien connaître et souvent une réanimation intense La deuxième phase de cicatrisation peut intégrer des modalités thérapeutiques modernes notamment l'oxygénothérapie hyperbare et la cicatrisation par pression négative utilisant le système VAC. Pour les pertes de substances étendues le recours à une chirurgie plastique reconstructrice est nécessaire.

### Etat des connaissances actuelle sur le sujet

- Il s'agit d'une infection grave mettant en jeu le pronostic vital dans l'immédiat et le pronostic fonctionnel à long terme;
- L'élément pronostic majeur reste la rapidité et la qualité de la prise en charge initiale;
- À distance plusieurs possibilités gestes de recouvrement cutané sont décrites.

### Contribution de notre étude à la connaissance

- Rapporter l'expérience d'une équipe tunisienne en matière de gangrène périnéales;
- Actuellement le débridement chirurgical classique et la fistulisation dirigée semble insuffisante et associée à une lourde morbidité et durées d'hospitalisations longues. Les moyens modernes notamment l'oxygénothérapie hyperbare et la vaco thérapie permet d'améliorer les résultats, diminuer les délais de cicatrisation;
- La prise en charge ne se conçoit que dans le cadre d'une prise en charge multi disciplinaire et une approche globale du malade.

## Conflits d’intérêts

Les auteurs ne déclarent aucun conflit d'intérêts.
